# Data on expectations, perceived quality, satisfaction with hospital care and financial ability of patients who suffer from acute and chronic respiratory diseases, in Central Greece

**DOI:** 10.1016/j.dib.2020.105564

**Published:** 2020-04-17

**Authors:** P. Koutsimpou, K.I. Gourgoulianis, A. Economou, V. Raftopoulos

**Affiliations:** a Municipality of Volos, University of Thessaly Medical School, Larissa, Greece; b University of Thessaly Medical School, Larissa, Greece; c Department of Economics, University of Thessaly, Greece; d Hellenic National Public Health Organization, Athens, Greece

**Keywords:** Patients’ satisfaction, Quality of care, Patients’ expectations, Respiratory diseases, Financial ability

## Abstract

The research article presents the data collected from a questionnaire based survey that aimed to evaluate patients’ expectations, perceived quality, satisfaction with hospital care and financial ability of 202 hospitalized patients suffering from acute or chronic respiratory diseases. The anonymous and self-completed questionnaire was divided in two parts. The first part included questions to elicit information on social and demographic characteristics (gender, age group, education level, categorization of respiratory disease, evaluation of the current hospitalization, nationality and way of living with). The second part included the 26-items Elderly Patient Satisfaction Scale and the 12-items Financial Ability Scale, which are validated in the Greek language with a high internal consistency. Data were collected from February 2016 to December 2018.

Specifications table

**Table:** 

Subject	Pulmonary and Respiratory Medicine
Specific subject area	Patients suffering from chronic and acute respiratory diseases
Type of data	Tables, Word files
How data were acquired	Questionnaire based survey
Data format	Raw, analyzed, descriptive
Parameters for data collection	Permission to carry out the research in the hospitals was provided by the Scientific Councils of the Public Hospitals in which the participants were hospitalized. A written consent was obtained from all the patients. The anonymity of the patients was guaranteed. They were given an introductory and information sheet for the purpose of the research and were informed that their participation was voluntary and that they were free to withdraw at any time without any consequence.
Description of data collection	An anonymous and self-completed questionnaire has been distributed to a sample of 202 hospitalized patients suffering from acute or chronic respiratory diseases.
Data source location	Larissa, Greece
Data accessibility	Data are hosted with the article

## Value of the data

• The data can be used for the evaluation of the expectations, perceived quality, satisfaction with care, and financial ability of patients who suffer from acute and chronic respiratory diseases in Central Greece.
• The data can be used from other researchers for comparison in different countries. These data add value to patient care especially in countries in which harsh austerity measures were enacted.
• The questionnaire and the validated scales can be used in other studies for the validation and cultural adaptation in their language and for benchmarking reasons [1,2].
• The data can be used for the improvement of the quality of care provided to patients with acute and chronic respiratory disease. Furthermore, the data are valuable for the development of a national policy for quality assurance of care provided to patients who suffer from respiratory diseases and for the improvement of their financial ability.

## 1 Data description

The dataset in this research article describes the data from 202 (57.4% men) hospitalized patients suffering from acute or chronic respiratory diseases.

Raw data of the questionnaire include patients’ responses (Supplementary Excel file format) to its items. The questionnaire used in the survey is included in a separated file (Supplementary Word file “Questionnaire”). The labels in the raw data file (Supplementary Excel file format) are in accordance with the items of the questionnaire. The labeling of the variable in the Excel file corresponds to the variable at the “questionnaire”. Mean scores have been calculated and the relevant labels are in the Excel file. The “questionnaire” included 3 sections: Section 1 includes 7 social and demographic characteristics (gender, age group, education level, categorization of respiratory disease, evaluation of the current hospitalization, nationality and way of living with) that are presented in Table 1, showing the frequencies and the percentages of their answers. The section 2 includes the 12-items Financial Ability Scale (FAS) and the section 3 the 26-items Elderly Patient Satisfaction Scale (EPSS). The patients were called to reply three times at the same items: one for rating their expectations, one for evaluating perceived quality of care and one for assessing their level of satisfaction with hospital care.

**Table 1:** Sociodemographic characteristics of the patients who participated in the survey.

Variable	*Ν*	%
**Gender**		
Men	116	57.4
Women	86	42.6
**Age group**		
<65	66	32.7
>65	136	67.3
**Education**		
Illiterate	26	12.9
Some primary	44	21.8
Primary	82	40.6
Secondary	31	15.3
Tertiary	17	8.4
MSc/PhD	2	1.0
**Respiratory disease**		
Chronic	111	55.0
Acute	91	45.0
**Current hospitalization**		
Worse compared to the previous hospitalizations	22	10.9
As good as the previous hospitalizations	86	42.6
Better compared to the previous hospitalizations	27	13.4
**Nationality**		
Greek	198	98.0
Other	4	2.0
**Living with**		
Family	172	85.1
Partner	3	1.5
Institution	1	0.5
Relatives	1	0.5
Parents	2	1.0
Alone	23	11.4

The mean scores of the patients at the 26 statements of the expectations, perceived quality and satisfaction with care scales as well as the mean financial ability of the participants are presented in Table 2. High scores indicate high expectations, perceived quality, satisfaction with care and financial ability. Separated comparisons of the differences between the two genders (Table 3), between age groups (Table 4), between persons with different education level (Table 5), between persons with chronic and acute respiratory diseases (Table 6) and between patients’ rating of the current hospitalization compared with others in the past (Table 7) regarding their mean expectations, perceived quality and satisfaction with care scores and financial ability have been performed. Table 8 shows the spearman correlation coefficients of the expectations, perceived quality, satisfaction with care and financial ability scores.

**Table 2:** Descriptive characteristics of EPSS and FAS.

	Expectancies	Perceived quality	Satisfaction	Financial ability
Mean	3.78	5.33	4.83	2.25
Median	3.84	5.50	4.84	2.00
Standard deviation	.43	.61	.52	0.82
Variance	.18	.38	.27	0.68
Min	2.46	3.35	2.62	1.00
Max	4.73	6.46	6.31	4.75
Range of scoring	0–5	0–7	0–7	1–5

**Table 3:** Differences between men and women regarding mean expectations, perceived quality and satisfaction with care scores.

Scale	Gender	*N*	Mean	SD	*p*-value
Expectations	Men	116	3.78	.40	0.842
	Women	86	3.77	.47	
Perceived quality	Men	116	5.34	.59	0.767
	Women	86	5.32	.65	
Satisfaction	Men	116	4.88	.52	0.153
	Women	86	4.77	.51	
Financial ability	Men	116	2.31	.82	0.200
	Women	86	2.16	.82

**Table 4:** Differences between age groups regarding mean expectations, perceived quality and satisfaction with care and financial ability scores.

Scale	Age group	*N*	Mean	SD	*p*-value
Expectations	<65	66	3.88	.39	0.025
	>65	136	3.73	.44	
Perceived quality	<65	66	5.53	.53	0.001
	>65	136	5.23	.63	
Satisfaction	<65	66	4.80	.46	0.563
	>65	136	4.85	.55	
Financial ability	<65	66	2.43	.88	0.029
	>65	136	2.16	.78

**Table 5:** Differences between persons with different education level regarding mean expectations, perceived quality and satisfaction with care and financial ability scores.

Scale	Education	*N*	Mean	SD	*p*-value
Expectations	Illiterate	26	3.68	.51	0.164
	Some primary	44	3.74	.41	
	Primary	82	3.74	.46	
	Secondary	31	3.91	.32	
	Tertiary	19	3.94	.29	
Perceived quality	Illiterate	26	5.09	.70	0.001
	Some primary	44	5.26	.55	
	Primary	82	5.25	.65	
	Secondary	31	5.64	.45	
	Tertiary	19	5.70	.35	
Satisfaction	Illiterate	26	4.82	.67	0.134
	Some primary	44	4.87	.51	
	Primary	82	4.90	.52	
	Secondary	31	4.80	.44	
	Tertiary	19	4.53	.32	
Financial ability	Illiterate	26	2.04	.85	<0.001
	Some primary	44	2.10	.71	
	Primary	82	2.02	.60	
	Secondary	31	2.55	.75	
	Tertiary	19	3.41	.92

**Table 6:** Differences between persons with chronic and acute respiratory diseases regarding mean expectations, perceived quality, satisfaction with care and financial ability scores.

Scale	Respiratory disease	*N*	Mean	SD	*p*-value
Expectations	Chronic	111	3.65	.50	<0.001
	Acute	91	3.93	.23	
Perceived quality	Chronic	111	5.02	.65	<0.001
	Acute	91	5.71	.27	
Satisfaction	Chronic	111	4.95	.56	<0.001
	Acute	91	4.69	.42	
Financial ability	Chronic	111	2.01	.65	<0.001
	Acute	91	2.54	.91

**Table 7:** Differences between patients’ rating of the current hospitalization compared with others in the past and mean expectations, perceived quality, satisfaction with care and financial ability scores.

Scale	Current hospitalization	*N*	Mean	SD	*p*-value
Expectations	Worse compared to the previous hospitalizations	22	3.93	.25	0.036
	As good as the previous hospitalizations	86	3.67	.47	
	Better compared to the previous hospitalizations	27	3.66	.44	
Perceived quality	Worse compared to the previous hospitalizations	22	5.69	.34	<0.001
	As good as the previous hospitalizations	86	5.11	.62	
	Better compared to the previous hospitalizations	27	5.09	.67	
Satisfaction	Worse compared to the previous hospitalizations	22	4.59	.44	0.008
	As good as the previous hospitalizations	86	4.89	.49	
	Better compared to the previous hospitalizations	27	5.01	.46	
Financial ability	Worse compared to the previous hospitalizations	22	2.57	1.08	0.010
	As good as the previous hospitalizations	86	2.19	.74	
	Better compared to the previous hospitalizations	27	1.88	.56

**Table 8:** Correlation between expectations, perceived quality, satisfaction with care and financial ability scores.

Scale	Perceived quality	Satisfaction	Financial ability
Expectations	.842 (*p*<0.001)	.245 (*p*<0.001)	.230 (*p*<0.001)
Perceived quality		.073 (*p* = 0.299)	.332 (*p*<0.001)
Satisfaction			−0.193 (*p* = 0.006)

## 2 Experimental design, materials, and methods

An anonymous and especially designed questionnaire was used to explore patients’ expectancies, perceived quality of care provided and satisfaction with hospital care, as well as their financial ability. The patients were recruited on the basis of their availability and their willingness to participate. A written informed consent was obtained from all the patients. The anonymity of the patients was guaranteed. They were given an introductory and information sheet about the purpose and the aim of the research and were informed that their participation was voluntary and that they were free to withdraw at any time without any consequence. The data collection has been conducted from February 2016 to December 2018 in one University hospital in the Central Greece. The eligible patients have been approached by the researcher.

The questionnaire was administered in the Greek language. The first part included questions to elicit information on social and demographics. The second part included the FAS and the EPSS which are validated in the Greek language with a high internal consistency [1,2].

The EPSS contains 26 statements that evaluate: (1) the patients’ expectations in terms of what patients expect from their hospital care (they were called to answer to a 6-likert scale ranging from 0: indifferent to 5: strongly agree), (2) the patients’ perceived quality of hospital care that assessed what they consider as quality of care components (they were called to answer to a 8-likert scale ranging from 0: indifferent to 7: very important) (3) the patients’ satisfaction with hospital care that consisted of the same 26 statements asking from the patients to answer how they feel with the care provided (they were called to answer to a 8-likert scale ranging from 0: indifferent to 7: very satisfied). In this research, patients’ expectations, perceived quality and satisfaction with hospital care were measured within the context of at least three days of hospitalization. The FAS contains 12 items that assess the financial ability of the patients as an indirect measure of the impact of economic crisis on their financial status. The participants were called to answer to each question (how do you rate your ability to) by using a 5-point Likert scale (very good, good, moderate, little, no ability).

All the items were coded and scored, and the completed questionnaires were included in the data analysis set. IBM-SPSS-25 [3] was used to analyze the data.

Supplementary data associated with this article can be found in the online version at

## CRediT authorship contribution statement

**P. Koutsimpou:** Conceptualization, Data curation, Methodology, Validation, Writing - original draft, Writing - review & editing. **K.I. Gourgoulianis:** Conceptualization, Supervision. **A. Economou:** . **V. Raftopoulos:** Conceptualization, Methodology, Validation, Writing - original draft, Writing - review & editing.

## Appendix Supplementary materials

Image, application 1

Image, application 2

Image, application 3
