# Supplementary material for: Data on expectations, perceived quality, satisfaction with hospital care and financial ability of patients who suffer from acute and chronic respiratory diseases, in Central Greece
Source: Data Brief. 2020 Apr 17;30:105564. doi: 10.1016/j.dib.2020.105564 (PMC7182669; doi:10.1016/j.dib.2020.105564)
Supplement: Supplementary file 2 [file mmc2.docx]

**QUESTIONNAIRE**

1. **GENDER**

Man (1)

Woman (2)

1. **Age Group**

<65 (1)

>65 (2)

1. **EDUCATION**

Illiterate (1) Some primary (2) Primary (3) Secondary (4)

Tertiary (5) MSc/Phd (6)

1. **MARITAL STATUS**

Single (1) Married (2) Divorced (3)

Widow (4) Cohabitation (5)

1. **NATIONALITY**

Greek (1) other (2)

1. **LIVING WITH**

My family (1) Partner (2) Institution (3)

Relatives (4) Parents (5) Alone (6)

**7. DIAGNOSIS AT THE CLINIC**

Chronic respiratory disease (1) Acute respiratory disease (2)

**8.** **THE PRESENT HOSPITALIZATION IN RELATION TO OTHERS:**

Worse compared to the previous hospitalizations (1)

As Good as the previous hospitalizations (2)

Better compared to the previous hospitalizations (3)

**Section 2: Financial Ability Scale (FAS)**

| How do you rate your ability |  | | | | |
| --- | --- | --- | --- | --- | --- |
|  | **NO ABILITY** | **LITTLE** | **MODERATE** | **GOOD** | **VERY GOOD** |
|  | **1** | **2** | **3** | **4** | **5** |
| 1.To buy basic goods (supermarket) |  |  |  |  |  |
| 2. To spend money for the house maintenance |  |  |  |  |  |
| 3. To spend money for heating |  |  |  |  |  |
| 4. To buy clothes and shoes |  |  |  |  |  |
| 5. To amuse themselves (entertainment, trips) |  |  |  |  |  |
| 6. To cover the expenses for paying bills |  |  |  |  |  |
| 7. To cover the expenses for taxation |  |  |  |  |  |
| 8. To share the expenses of their relatives (children, grandchildren) |  |  |  |  |  |
| 9. To cover the expenses for their medicines |  |  |  |  |  |
| 10. To cover the expenses for other therapies (physio, dental etc) |  |  |  |  |  |
| 11.To save money |  |  |  |  |  |
| 12. To have a housekeeper |  |  |  |  |  |

**Section 3: Expectations Perceived quality Satisfaction Scale**

|  | EXPECTANCIES |  | PERCEIVED QUALITY |  |  | **SATISFACTION** |
| --- | --- | --- | --- | --- | --- | --- |
|  |  |  |  |  |  |  |
| **5** | STRONGLY AGREE | **7** | EXTREMELY IMPORTANT |  | **7** | EXTREMELY SATISFIED |
| **4** | AGREE | **6** | VERY IMPORTANT |  | **6** | VERY SATISFIED |
| **3** | DISAGREE | **5** | MODERATELY IMPORTANT |  | **5** | MODERATELY SATISFIED |
| **2** | STRONGLY DISAGREE | **4** | ALMOST IMPORTANT |  | **4** | ALMOST SATISFIED |
| **1** | NEUTRAL | **3** | SLIGHTLY IMPORTANT |  | **3** | SLIGHTLY SATISFIED |
| **0** | INDIFFERENT | **2** | ΝΟ IMPORTANT |  | **2** | ΝΟ SATISFIED |
|  |  | **1** | NEUTRAL |  | **1** | NEUTRAL |
|  |  | **0** | INDIFFERENT |  | **0** | INDIFFERENT |

|  |  |  |  |
| --- | --- | --- | --- |
|  | ***I expect*** | ***How important is it for you?*** | ***How do you feel?*** |
|  |  |  |  |
| 1. Have food variety. |  |  |  |
| 1. Serve hot food in a nice way |  |  |  |
| 1. Food to be tasty |  |  |  |
| 1. The room is kept clean; clean toilets and the sheets are often changed |  |  |  |
| 1. Observe visiting hours |  |  |  |
| 1. Feeling that the Department is well managed |  |  |  |
| 1. Feeling safe in the hospital |  |  |  |
| 1. Have enough nursing staff in the night shift |  |  |  |
| 1. Have a comfortable room and a comfortable bed |  |  |  |
| 1. Have a good and quiet sleep without interruption by staff noises |  |  |  |
| 1. To feel that medical doctors and nurses are not indifferent when I suffer (from stress, pain). |  |  |  |
| 1. Feeling that medical doctors and nurses generally respect my personality |  |  |  |
| 1. Medical doctors and nurses inspire me so much that I can discuss with them my very own problems |  |  |  |
| 1. Medical doctors reveal me the whole truth about my health |  |  |  |
| 1. I can always find a doctor when I need to |  |  |  |
| 1. I can always find a nurse when I need to |  |  |  |
| 1. The staff talks to me in a polite way |  |  |  |
| 1. When medical doctors examine my body, they respect me |  |  |  |
| 1. Feeling that nurses know their job well (when they give drugs, take blood) |  |  |  |
| 1. Staff does not discuss my case in front of third parties or other patients |  |  |  |
| 1. The staff asks for my consent before they do any intervention |  |  |  |
| 1. Medical doctors explain to me the diagnosis and medical examination results in a simple and understandable way |  |  |  |
| 1. Medical doctors advice me how to maintain healthy |  |  |  |
| 1. Medical staff cares me without asking for money |  |  |  |
| 1. The staff respects the presence and interest of my relative |  |  |  |
| 1. Medical doctors had enough time to take a full health history that would be useful for a correct diagnosis |  |  |  |
